# Residues of Deltamethrin in Pine Needles and Pine Nuts of Catalonia (Spain)

**DOI:** 10.3390/molecules28248050

**Published:** 2023-12-12

**Authors:** Marina Bellot, Anna Teixidó, Antoni Torrell, Neus Aletà, Cristian Gómez-Canela

**Affiliations:** 1 Department of Analytical and Applied Chemistry, School of Engineering, Institut Químic de Sarrià-Ramon Llull University, Via Augusta 390, 08017 Barcelona, Spain; marina.bellot@iqs.url.edu; 2 Multifunctional Forest Management Program, Forest Science and Technology Centre of Catalonia (CTFC), Ctra. St. Llorenç de Morunys, Km 2, 25280 Solsona, Spain; anna.teixido@irta.cat; 3 Department of Climate Action, Food and Rural Agenda, Forestal Catalana S.A., Dr. Roux 80, 08017 Barcelona, Spain; atorrells@gencat.cat; 4 Agroforetsry Group, Fruit Tree Program, Institute of Agrifood Research and Technology (IRTA), Torre Marimon, 08140 Caldes de Montbui, Spain; neus.aleta@irta.cat

**Keywords:** pine needle, nut, deltamethrin, gas chromatography

## Abstract

In recent years, recurrent droughts have weakened stone pine (*Pinus pinea*) forests and facilitated the emergence of harmful pests and diseases, including the *Leptoglossus occidentalis*. The production of stone pine nuts has declined over the past five years. To control this hemipteran pest, a synthetic pyrethroid insecticide called deltamethrin is being tested. However, it is necessary to estimate the residue left by these treatments in forest stands. Therefore, a fast and robust analytical procedure was developed based on QuEChERS clean-up extraction, followed by gas chromatography coupled with an electron capture detector. This optimized method can detect residual concentrations of deltamethrin in pine nuts and pine needles up to 0.1 and 6 μg kg^−1^, respectively, with a limit of quantification of 0.4 and 20 μg kg^−1^. Great recoveries (between 84 and 102%) were obtained for both matrices, and no matrix effect was observed. The results showed that two weeks after spraying, the deltamethrin content in the needles of stone pines decreased by up to 75%, and after nine months, its presence was like that of nontreated trees.

## 1. Introduction

The stone pine, *Pinus pinea* L., is a woody species, very common in the Iberian forests on sandy soils, occupying over 600,000 hectares in the Iberian Peninsula either as scattered trees, members of the Mediterranean mixed forest, or forming monospecific stands [1,2]. However, the species is currently facing the consequences of emerging pests, whose expansion is mainly facilitated by recurrent droughts in recent years. One of the most damaging and concerning pests is the Western conifer seed bug (*Leptoglossus occidentalis* Heidemann), identified in Catalonia, Spain, in 2003 [3]. These bedbugs suck vegetative and reproductive organs of pines and destroy cones of different ages before they reach maturity. Pine nuts are highly valued for their nutritional qualities [2,4] but their production has dramatically decreased over the last five years due to the actions of these pests [5,6,7]. To control this pest, deltamethrin (DLT), a synthetic pyrethroid ester with broad-spectrum acaricidal and insecticidal activity, has been tested in the field with very good results. However, the use of pesticides raises concerns about their slow rates of degradation, toxicity, and the potential for accumulation in remote areas of the world [8]. They may enter the atmosphere through several processes: from pesticide formulations applied over farming areas and forested landscapes [9], volatilized from the surface of soil, water, and plants [8], or the incineration of waste materials treated with pesticides [10], among others. The World Health Organization (WHO) considers pesticide and veterinary drug residues in foodstuffs to be of utmost importance in global public hygiene, posing potential hazards to food safety. In this context, the Food and Agriculture Organization (FAO)/WHO/Codex Alimentarius Commission (CAC) sets maximum residue limits (MRLs) for more than 170 pesticide and veterinary drug residues in over 300 varieties of agricultural products and foods [11]. In the case of pine nuts, the European commission has established a maximum allowed value of deltamethrin of 20 μg kg^−1^ (Reg. (EU) 2018/832) [12]. However, there is no legislation regarding the concentration of this pesticide in Pinus stands. Therefore, to the best of our knowledge, no study has reported a robust method capable of detecting and quantifying residue levels of deltamethrin in pine needles and pine nuts. Most of the projects that have studied pyrethroid residues have reported methodologies based on gas chromatography (GC) coupled to a mass spectrometry detector (MS) or electron capture detector (ECD) [13,14]. These substances have high molar mass, high boiling point, and are also very electronegative due to the actions of Bromine atoms in the molecules. Additionally, the complex matrix (pine needles and pine nuts) with many resins, wax [8], lipids (nuts), and chlorophylls (needles) requires a clean-up step to minimize the matrix effect in the analysis of the target analyte [8,13,14,15]. A methodology capable of detecting and quantifying traces of pesticides in environmental samples, such as pine needles or pine nuts, could be very helpful for environmental monitoring (pesticide residues assessment or ecological impact assessment) and for food safety and agricultural practices monitoring. Thus, the present study aims to develop and optimize a fast and robust analytical procedure capable of determining low residue levels of deltamethrin in pine needles and pine nuts through GC-ECD analysis. To check the efficiency of the method, monitoring was carried out in a trial planned to evaluate the deltamethrin’s effectiveness in controlling Western conifer seed bug damages, which was designed in a productive stand of Stone pine.

## 2. Results and Discussion

### 2.1. Optimization of the Extraction Procedure

**Figure 1:**
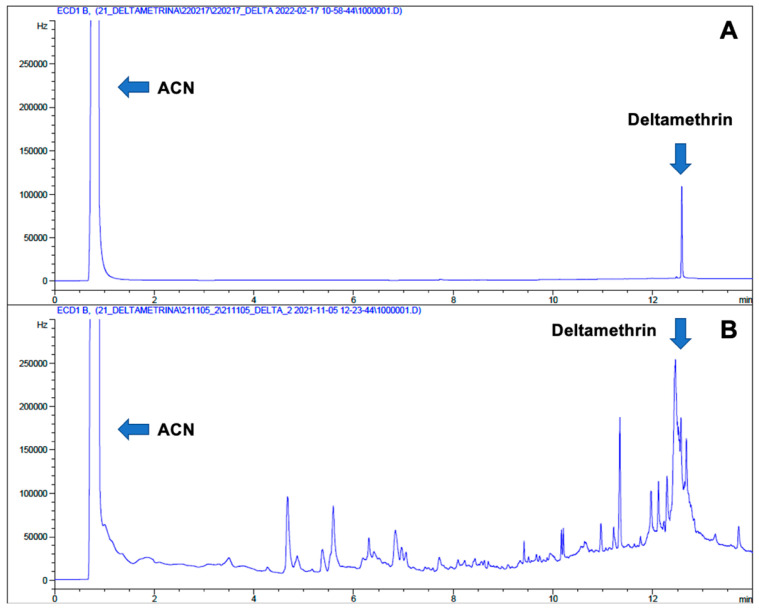
Chromatogram of deltamethrin the standard (**A**), where the elution peak of solvent (ACN) and deltamethrin can be observed. Chromatogram of a QC of pine needles analysis using Method A (**B**).

The efficiency of the analytical procedure was calculated through the recovery method. Low recoveries were obtained in all procedures based on ultrasound-assisted extraction (UAE) (Method A). Figure 1A displays a standard of DLT at 100 μg L^−1^ (tR = 12.6 min) in comparison with a spiked sample (QC) of a pine needle (Figure 1B) using Method A. In the case of the spiked pine needle, a lot of chromatographic peaks are observed due to matrix interferences. However, in the spiked pine nuts samples (QCs), no interferences were observed, and the peak of DLT was clearly distinguished. Based on these results, it can be assumed that the peaks observed in pine needles samples could be due to the large number of chlorophylls present in the tree foliage. These molecules have four nitrogen atoms in their structure, giving a higher response in ECD due to their high electronegative character. In pine nuts samples, no interfering peaks were observed since chlorophylls are not typically found in these samples. Moreover, solvent blanks (without a sample but spiked) presented good recoveries above 70% (with all the tested cartridges), indicating that no matrix effect was occurring.

On the other hand, for the spiked pine needles and pine nuts samples using Method B, recoveries were above 80% for both matrices. This methodology represented an improvement in the efficiency of the method, being also faster, cheaper, and easier to perform than the other methodologies tested. Moreover, this methodology was more environmentally friendly since it used a lower amount of solvent than that used in Method A. For all these reasons, Method B was selected to perform all the analyses in real samples.

### 2.2. Quality Parameters

A great correlation was obtained (R^2^ = 0.998) for the range between 0.25 to 1000 μg L^−1^ (0.5 μg kg^−1^ to 2000 μg kg^−1^) of DLT in ACN. Moreover, the instrumental detection limit was 3 pg, while the MDLs for pine needles and pine nuts were 6 μg kg^−1^ and 0.1 μg kg^−1^, respectively. Limits of quantification were assessed at 20 μg kg^−1^ (pine needles) and 0.4 μg kg^−1^ (pine nuts). On the other hand, precision was calculated as intra-day and inter-day precision, obtaining results of 0.9% and 9.8%, respectively.

As observed in Table 1, for pine needles, the average recovery was 84.9% for the lower level and 83.8% for the higher level, while for pine nuts, the mean recovery at the low level was 101.7%, and 87.3% for the higher level. Finally, no matrix effect was observed in either pine needles or pine nuts (ME pine needle = 83 ± 2%, ME pine nuts = 80 ± 1%).

### 2.3. Residues of Deltamethrin in Pine Needles and Pine Nuts

**Figure 2:**
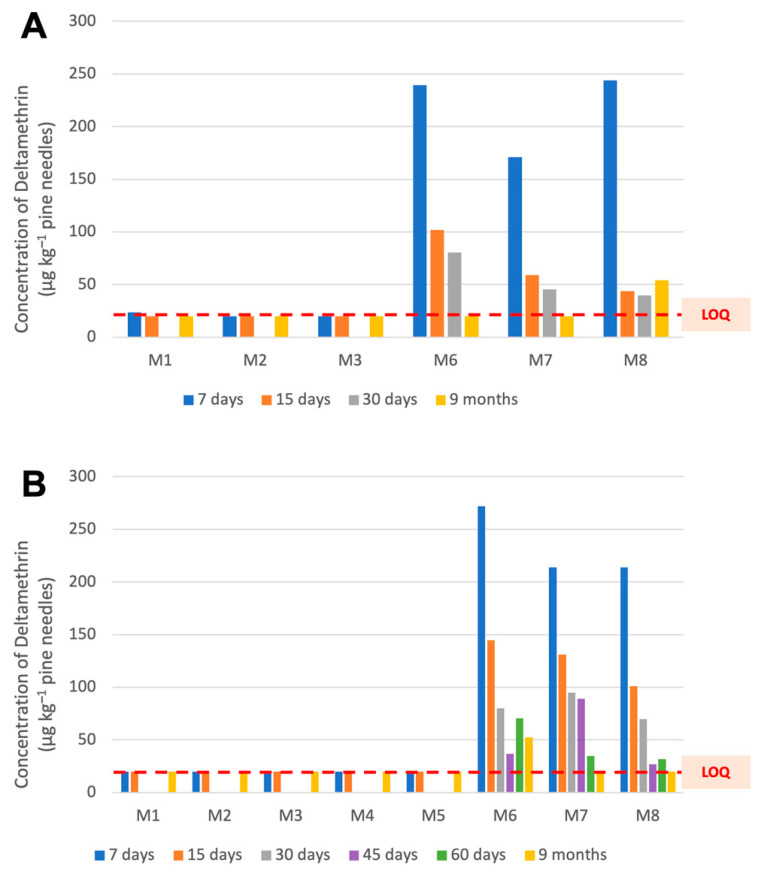
Deltamethrin concentration observed in pine needles in the 2021 crop season (**A**) and 2022 (**B**). Red discontinuous line indicates values below the limit of quantification (20 μg kg^−1^).

Results allowed a clear differentiation between the two treatments, treated by the insecticide (TR) and non-treated (NT), in both crop seasons. Throughout the first growing season, from August 2021 to April 2022, all residue contents were under the limit of quantification (LOQ) of the method. During this period, the TR pines (needles), which started 7 days after application with a DLT content of about 200 μg kg^−1^, lost more than 65% of the insecticide after 30 days (Figure 2A). The decrease in concentration in samples could be explained as a result of the drift provoked by different weather conditions, such as rain or wind. 

However, this concentration was still above 20 μg kg^−1^, the limit established for pine nut consumption by the European Pesticide Data Base (2021), which was considered in this work since there is no such limit for pine needles. On the other hand, after nine months of insecticide application, needles did not show differences between both TR and NT treatments, and the residue found was lower than 20 μg kg^−1^. Only M8, from the TR zone, showed slightly higher levels than the samples taken 30 days after insecticide application. All concentration levels observed in the first crop season are summarized in Table S1.

Results issued from the second crop season (2022) followed a similar pattern to those of 2021 for both treatments (Figure 2B). In the TR zone, the reduction in DLT content in needles was progressive over time, but there was a discordant value at 60 days (M6 in Table S2). However, after nine months in field conditions, needles did not show differences between both TR and NT treatments, and in both cases, the amount found was lower than 20 μg kg^−1^. The results of the second campaign are summarized in Table S2.

**Figure 3:**
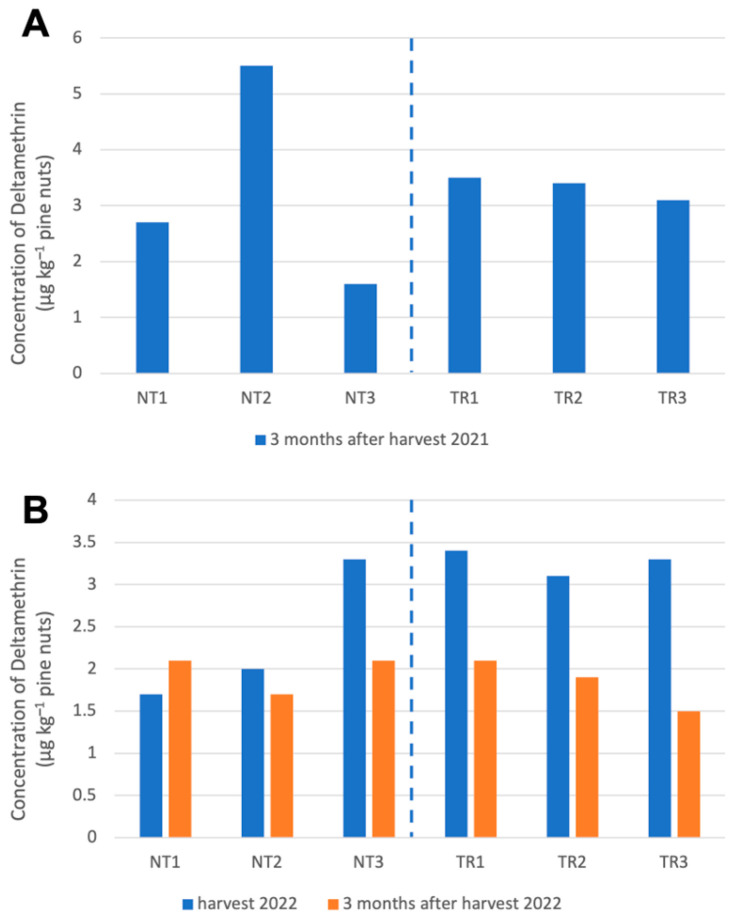
Deltamethrin concentration in pine nuts for the harvest years 2021 (**A**) and 2022 (**B**). In the left part are samples from non-treated areas (NTs), and in the right part are samples from treated areas (TRs).

In the analyses of pine nuts performed, both three months after harvest (2021 and 2022) and just after harvest (2022), only traces of DLT were found, with values well below the permitted limit for pine nut consumption (European Pesticide Data Base, 2021) (Figure 3). 

Deltamethrin residue showed a similar evolution pattern during the two growing seasons for both pine nuts and needles. However, the concentration in the nut matrix was much lower than in the needles. The application of DLT in the control of *Leptoglossus occidentalis* does not imply any limitation for the consumption of pine nuts; in all the determinations carried out, the residue level was below the limits established by the relevant European control administration. The difference between the results on nuts at harvest and those at three months was minimal and often below the limit of quantification of the method (0.4 μg kg^−1^). In addition, the harvest of this species is not marketed until well into the spring of the following year, which separates the application of the product until its consumption by more than 9 months. 

For needles, it has been verified in 2021 that 9 months after application on trees, residues in the field have dropped to very low levels, like those of NT areas. Data from 2022 showed that at 60 days, the residue of DLT was like those after 9 months in 2021, and a considerable decrease was observed after 9 months. It should be borne in mind that DLT is a broad-spectrum insecticide and widely used in the production of many vegetables and fruits, and its transport could be expected by the wind, particularly in windy areas like Alt Empordà (Girona-Spain).

### 2.4. Residues of Deltamethrin in Other Food Matrices

In the last years, several studies have reported higher levels of deltamethrin residues in vegetables than the established MRLs. In 2021, Elgueta et al., in a study published about the analysis of multi-pesticide residues in fresh tomatoes, determined that 61% of the tomatoes cultivated in greenhouses in the Mediterranean region of Turkey contained many active substances, among which DLT was found [16]. Moreover, Salghi et al. reported DLT residues in tomatoes from greenhouses in Morocco higher than MRL (0.3 mg kg^−1^). In this case, 10% of the samples where above MRL, while the other samples were between 0.003 and 0.25 mg kg^−1^ [17]. Furthermore, Jallow et al. performed an analysis to monitor pesticide residues in commonly consumed fruits and vegetables in Kuwait, and they reported residues of DLT exceeding the MRLs in watermelons, apples, and grapes. Interestingly, deltamethrin was the second most detected pesticide (11% of the analyzed samples contained DLT) [18]. However, subsequent studies performed in Spain determined values below MRLs for deltamethrin in lettuce, spinach, and Swiss chard [19]. In any case, previously reported values in other matrices such as vegetables or fruits, are higher than the reported values in this study for pine nuts.

However, it is worth noting that the detection of DLT residues above the MRLs does not necessarily mean that the vegetables are unsafe for consumption. MRLs are set based on scientific data and risk assessments, and exceeding them does not always imply a health risk. However, it does indicate that the levels of DLT in those vegetables are higher than what is deemed acceptable by the regulatory authorities. In such cases, appropriate measures are taken to prevent further contamination, and the vegetables are withdrawn from the market.

## 3. Materials and Methods

### 3.1. Reagents and Chemicals

High-purity standard of deltamethrin (DLT) was supplied by Merck (Darmstadt, Germany). Acetonitrile (ACN) was supplied by Scharlab (Barcelona, Spain), while hexane (HX), dichloromethane (DCM), and acetone (CO), all of them Chromanorm^®^ grade, were supplied by VWR Chemicals (Leuven, Belgium). Moreover, QuEChERS kits were purchased from Phenomenex (Torrance, CA, USA). Florisil (Strata^®^, Phenomenex), ENVI 18 (Supelclean^®^, Supelco Analytical, Bellefonte, PA, USA), and HLB (OASIS^®^, Waters, Milford, MA, USA) cartridges were tested and evaluate for clean-up purposes. 

Stock solutions of DLT were prepared at 1 mg mL^−1^ in ACN, weighing 1.00 mg and diluting in 1 mL of solvent. This standard was kept at −20 °C in amber vials to prevent degradation. Calibration standards were prepared daily in ACN. 

### 3.2. Field Experimental Design 

**Figure 4:**
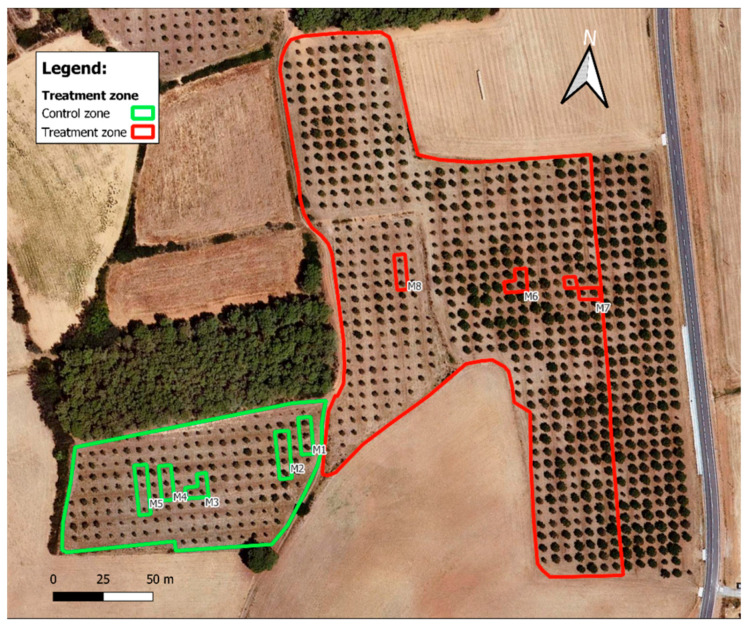
Location of the two zones of study. Treated pines (TRs, red) and untreated pines (NTs, green). In 2021’s crop season NT samples were collected from M1, M2, and M3, while in 2022’s crop season, NT samples were collected from all pointed samples (M1–M5).

In a productive 10-year-old *P. pinea* orchard, located in Arenys d’Empordà in Northeast Catalonia (Spain), two areas were delimitated to set up the trial to evaluate the efficacy of the insecticide, DLT 2.5% *w*/*v*, in controlling the pest. One area, represented by the red color in Figure 4, was treated with the pesticide (0.2 L/h) and labelled as treated pines (TRs), while the other area, represented by the green color, was non-treated and labelled as untreated pines (NTs). Each plot had a rectangular polygon shape and occupied at least two hectares to ensure representative sampling of pine needles and nuts from each treatment, as the farm is located in an area where persistent and strong wind blows (la ‘tramontana’) very often. All treatments were carried out in accordance with European and national regulations. Two treatments were applied in 2021—one in May, after the peak of adult presence of the post-winter generation, and the other at the end of July, applied after the peak of the insect harmful stages (adults or nymphs 2+) presence at the first annual generation. The population dynamics of the *Leptoglossus occidentalis* were monitored weekly in both TR and NT plots to carefully guide the timing of treatments. 

Two brands of deltamethrin-based products were applied, Deltaplant^®^ and Decis^®^, both with a declared security period of 30 days. The most predominant wind in this area is tramontane, a strong northern wind that prevailed in the region for centuries, shaping the landscape and influencing the local culture and way of life.

### 3.3. Sampling

Pine needle samples, weighing 15–20 g, were randomly collected from three identified pines (25–30 needles per tree) in both the TR and NT during two consecutive crop seasons (2021 and 2022). Table 2 shows the number of samples, needles, or pine nuts, established by each record. In the first year (2021), in the TR area, three samples of needles were collected at 7, 15, and 30 days after the last treatment and again at 9 months into 2022, resulting in four sampling times per treatment (Table S1). The three NT samples were taken: one in the center of the plot and the other two at the edges (first and third rows), trying to assess the effect of product drift in this windy area such as Alt Empordà (Figure 1). In this NT area, needles were removed only at 7 and 15 days and again at 9 months (Table S1). In the second crop season, 2022, the number of samples taken in the NT areas was five, with three in the middle of the plot and two at the edges (Figure 4); those from the TR area were three, as in the previous crop season. The sampling times in NT areas were twice, at 7 and 15 days (Table S2), while in TR, it increased to five in order to build a more detailed temporal decreasing pattern of DLT in pine needles; samples were collected at 7, 15, 30, 45, and 60 days from the last treatment. Moreover, samples were collected after 9 months (Table S2). All needle samples were wrapped in aluminum foil in the field and stored in glass containers at a low temperature of 4 °C until GC-ECD analysis.

In the case of pine nuts, 20 g of pine nut kernels were extracted from harvested cones of both TR and NT areas in 2021 and 2022 crop seasons. Ripe cones were dried in an oven at 40 °C to get them to open. Each sample of pine nuts came from cones randomly collected on 10 trees per treatment and distributed among three samples for analysis (Table 2). Pine nuts were stored in glass containers at fridge temperature until analysis. In 2021, pine nut residues were only analyzed once, at three months at harvest (February 2022), which was more than six months after the last treatment (6 analyses). In 2022, they were analyzed twice at harvest and three months later, as in the previous year (November 2022 and February 2023).

### 3.4. Deltamethrin Extraction

Different extraction and clean-up procedures were tested to optimize the determination of residual levels of deltamethrin in pine needles and pine nuts. The extraction procedures tested were selected based on their different mechanisms of extraction and retention. Initially, sonication or ultrasound-assisted extraction was evaluated for its ability to extract analytes from the matrix using various solvents. Subsequently, different stationary phases with varying degrees of retention for the clean-up step were evaluated. Florisil (Strata^®^, Phenomenex), ENVI 18 (Supelclean^®^, Supelco Analytical), and HLB (OASIS^®^, Waters) were all used to retain pesticides. Additionally, QuEChERS, a matrix-dispersive mode of solid-phase extraction, was tested. 

Both pine needles and pine nuts samples were ground in a grinder with a steel propeller till the size of the particles was close to 1 mm. Then, 5 g of the sample was placed in a 50 mL Falcon tube. Two different methods were tested: ultrasonic extraction (Method A) and QuEChERS (Method B). Four different organic solvents with different polarities, namely hexane (HX) (Method A1), acetonitrile (ACN) (Method A2), HX:ACN 50:50 (Method A3), HX:DCM 50:50 (Method A4), and HX:Acetone (CO) 50:50 (Method A5), were employed for ultrasonic extraction. Twenty-five milliliters of each solvent were placed in the Falcon tube containing the sample, and the mixture was sonicated in an ultrasonic bath for 30 min. After this period, the solvent was replaced and filtered through a cellulose filter by gravity into a round-bottom flask. This process was repeated thrice, and the final volume was 75 mL, with a total extraction time of 90 min. The round-bottom flask containing the solvent was then placed in a rotavap to reduce the solvent to almost dryness (about 2 mL). A clean-up step was then implemented to minimize interferences from the matrix, with three different cartridges containing different solid extractants (HLB, Florisil, and C18) tested. 

First, the cartridges were conditioned with 10 mL of the extraction solvent. Then, the remaining volume (≈2 mL) was charged into the cartridge and dried under vacuum for 10 min. The elution step was carried out using 10 mL of solvent (2 × 5 mL). The final extract was almost dried using a stream of nitrogen in a sample concentrator (Techne^®^, Fisher Scientific S.L., Madrid, Spain) and transferred to a 1.8 mL chromatographic vial. Finally, the samples were evaporated to dryness and reconstituted with 1 mL of ACN. 

On the other hand, in Method B, a kit containing 4 g MgSO_4_, 1 g NaCl, 1 g SCTD (sodium citrate tribasic dihydrate), and 0.5 g SCDS (sodium citrate dibasic sesquihydrate) was placed in each Falcon tube containing the sample. Then, 10 mL of ACN was added to the mixture and shaken vigorously for 1 min. The tubes were centrifuged at 4000 rpm for 5 min in an OrtoAlresa (Madrid, Spain) centrifuge. Afterwards, 5 mL of the supernatant was transferred to a 25 mL Falcon tube containing 0.9 g MgSO_4_ and 150 mg PSA (primary and secondary amine exchange material). The mixture was shaken manually for 30 s and centrifuged at 4000 rpm for 5 min. Finally, 1 mL of the supernatant was transferred to a chromatographic vial for the GC-ECD analysis. Figure S1 displays the summary of all the extraction methods tested.

### 3.5. GC-ECD Analysis

Deltamethrin analysis was performed by GC-ECD, using an Agilent 6890 N instrument (Santa Clara, CA, USA, EUA) coupled to an electron capture detector (ECD). The carrier gas was Helium (He) at a constant flow rate of 51 mL min^−1^. The injector temperature was set at 250 °C, and 2 μL were injected in splitless mode with a purge flow of 40 mL min^−1^. The GC column was HP-5MS (30 m × 0.32 mm × 0.25 μm), and the elution was performed using a gradient program. First, the column oven was set at 120 °C and held for 3 min. Then, the temperature increased at a rate of 20 °C min^−1^ to 310 °C and held for 8 min, resulting in a total run time of 20 min. The pressure was 21.76 psi. Regarding detector parameters, it was set at 350 °C, and the make-up gas (nitrogen) flow was fixed at 70 mL min^−1^.

### 3.6. Quality Assurance

The analytical method was evaluated in terms of quality, based on different parameters such as linearity, limit of detection, limit of quantification, inter- and intra- day precision, and recoveries. Regarding the linearity of the instrument methodology, five standards of pure DLT were prepared in ACN within the concentration range of 0.25 to 1000 μg L^−1^ (0.5–2000 μg kg^−1^). The instrumental detection limit (IDL) was assessed using the signal-noise ratio (S/N) of the less concentrated standard, considering a minimum S/N of 3. Precision was determined by analyzing the same standard three times on the same day (intra-day precision) and the same standard (100 μg L^−1^) for three consecutive days (inter-day precision).

Furthermore, for each extraction methodology assay, four replicates of quality controls (QCs) were prepared at two different levels (20 and 1000 μg kg^−1^) with non-treated samples of both matrices (pine needles and pine nuts). Figure S2 shows a GC-ECD chromatogram of a standard at 20 μg kg^−1^ (A), a QC of pine needle at 20 μg kg^−1^ (B), and pine nuts at 20 μg kg^−1^ (C). The analysis of QCs allowed the determination of other quality parameters. Methodological detection limit (MDL) and limit of quantification (LOQ) were evaluated using the S/N of QCs. Recoveries, expressed as a percentage, were determined by comparing the spiked concentration with the experimental concentration. Moreover, blank (no sample and non-spiked), solvent blank (no sample but spiked), and procedural blanks (with a sample but non-spiked) were studied in order to check that there was no cross-contamination. Finally, the matrix effect (ME) was assessed by comparing the peak area of DLT from the spiked samples (20 μg kg^−1^) with the peak area of the analyte from the standard solution used in the calibration curve. This parameter is an indicator of detection suppression or enhancement of the analytes.

## 4. Conclusions

A comprehensive optimization of a QuEChERS method followed by GC-ECD was performed for the identification and quantification of deltamethrin in pine needles and pine nuts. The analytical performance of the GC-ECD method provided high selectivity and sensitivity and enhanced identification capabilities. After the analysis of these samples, the study successfully differentiated between the two treatments: insecticide-treated (TR) and non-treated (NT), in both crop seasons. During the first growing season (August 2021 to April 2022), all residue concentrations for NT areas were below the limit of quantification (LOQ) of the method used. Initially, the TR pine needles had a DLT content of approximately 200 μg kg^−1^, but after 30 days, they lost more than 65% of the insecticide. After nine months of insecticide application, there were no significant differences between the TR and NT treatments in terms of residue levels in the pine needles, and the amount found was below 20 μg kg^−1^, for all samples except one of them. In the second crop season (2022), the results followed a similar pattern to the previous season for both treatments (TR and NT). Therefore, it can be concluded that even though deltamethrin is considered a persistent pollutant, the residue in pine needles after a “short” period since the treatment were below the limit that the European administration established for pine nuts.

Finally, in the case of pine nuts, the application of deltamethrin in the control of *Leptoglossus occidentalis* implies a limitation of DLT residues for the consumption of pine nuts. In all the determinations carried out, the residue levels were below the limits established by the relevant European control administration.

## Figures and Tables

**Table 1:** Quality parameters of the optimized methodology for determining deltamethrin by GC-ECD.

Quality Parameters		Pine Needles	Pine Nuts
Limit of detection	Instrumental	3 pg	
	Methodological	6 μg kg^−1^	0.1 μg kg^−1^
Limit of quantification		20 μg kg^−1^	0.4 μg kg^−1^
Precision	Inter-day	0.9%	
	Intra-day	9.8%	
Recovery	Low level (20 μg kg^−1^)	84.9 ± 7%	101.7 ± 3%
	High level (1000 μg kg^−1^)	83.8 ± 5%	87.3 ± 2%
ME (20 μg kg^−1^)		83 ± 2%	80 ± 1%

**Table 2:** Position and number of samples taken in the field at each sampling point (NT: non treated area, TR: treated area).

Crop Season	Treatment	Needles			Pine Nut	
		Id. Figure 4.	Plot Location	Samples	Id. Figure 4	Samples
2021	NT area			3	NT1 NT2 NT3	3
		M1	First row	1		
		M2	Third row	1		
		M3	Centre of plot	1		
	TR area	M6 M7 M8	Centre of plot	3	TR1 TR2 TR3	3
2022	NT area			5	NT1 NT2 NT3	3
		M1	First row	1		
		M2	Third row	1		
		M3 M4 M5	Centre of plot	3		
	TR area	M6 M7 M8	Centre of plot	3	TR1 TR2 TR3	3

## Data Availability

Data are contained within the article and Supplementary Materials.

## Supplementary Materials

The following supporting information can be downloaded at: https://www.mdpi.com/article/10.3390/molecules28248050/s1, Table S1: Residues quantified on pine needle samples at different intervals of time after an aerial application of Deltamethrin (0.2 L/ha) in Arenys d’Empordà (Girona-Spain) in 2021’s crop season; Table S2: Residues quantified on pine needle samples at different intervals of time after an aerial application of Deltamethrin (0.2 L/ha) in Arenys d’Empordà (Girona-Spain) in 2022’s crop season; Figure S1: Detailed steps of the extraction and clean-up procedures under study. Figure S2. GC-ECD chromatogram of an standard at 20 μg kg^−1^ (A), an a QC of pine needle at 20 μg kg^−1^ (B) and pine nuts at 20 μg kg^−1^ (C).
