# Supplementary material for: Residues of Deltamethrin in Pine Needles and Pine Nuts of Catalonia (Spain)
Source: Molecules. 2023 Dec 12;28(24):8050. doi: 10.3390/molecules28248050 (PMC10745627; doi:10.3390/molecules28248050)
Supplement: Supplementary file 1 [file molecules-28-08050-s001.zip › molecules-2742623-supplementary.pdf]

Supplementary material

Development of an analytical method based on QuEChERS and GC-ECD for the determination of deltamethrin residue in pine needles and pine nuts

Marina Bellot[1], Anna Teixidó[2], Antoni Torrell[3], Neus Aletà[4], Cristian Gómez-Canela[1*]

Table S1. Residues quantified on pine needle samples at different intervals of time after an aerial application of Deltamethrin (0,2 L/ha) in Arenys d'Empordà (Girona-Spain) in 2021's crop season.

Treatments	Sample code	Time	Weight (g)	Deltamethrin (µg kg^{-1})
Non treated area (NT)	M1	7 days	5.0178	23.8
		15 days	5.1405	< 20 (< LOQ)
		9 months	5.0272	< 20 (< LOQ)
	M2	7 days	5.1276	< 20 (< LOQ)
		15 days	5.1578	< 20 (< LOQ)
		9 months	4.9785	< 20 (< LOQ)
	M3	7 days	5.1822	< 20 (< LOQ)
		15 days	4.9993	< 20 (< LOQ)
		9 months	5.0450	< 20 (< LOQ)
Treated area (TR)	M6	7 days	5.0605	239.6
		15 days	5.0299	102.0
		30 days	5.0690	80.5
		9 months	5.0860	< 20 (< LOQ)
	M7	7 days	5.0163	170.9
		15 days	5.1503	59.2
		30 days	5.0313	45.6
		9 months	4.9571	< 20 (< LOQ)
	M8	7 days	4.9640	244.1
		15 days	5.0971	43.9
		30 days	5.0629	39.6
		9 months	4.9934	54.0

Table S2. Residues quantified on pine needle samples at different intervals of time after an aerial application of Deltamethrin (0,2 l/ha) in Arenys d'Empordà (Girona-Spain) in 2022's crop season.

Treatment	Sample code	Time	Weight (g)	Deltamethrin (µg kg^{-1})
Non treated area (NT)	M1	7 days	4.6737	< 20 (< LOQ)
		15 days	5.0201	< 20 (< LOQ)
		9 months	4.9960	< 20 (< LOQ)
	M2	7 days	5.0262	< 20 (< LOQ)
		15 days	5.0063	< 20 (< LOQ)
		9 months	5.0740	< 20 (< LOQ)
	M3	7 days	4.7130	< 20 (< LOQ)
		15 days	5.0582	< 20 (< LOQ)
		9 months	4.9748	< 20 (< LOQ)
	M4	7 days	5.0047	< 20 (< LOQ)
		15 days	4.9880	< 20 (< LOQ)
		9 months	5.0290	< 20 (< LOQ)
	M5	7 days	4.9941	< 20 (< LOQ)
		15 days	5.0131	< 20 (< LOQ)
		9 months	5.04273	< 20 (< LOQ)
Treated area (TR)	M6	7 days	5.0227	271.8
		15 days	4.9866	144.5
		30 days	5.0582	80.1
		45 days	5.0226	36.7
		60 days	4.9663	70.6
		9 months	5.0374	52.6
	M7	7 days	5.0044	213.9
		15 days	4.9911	131.2
		30 days	5.0060	95.0
		45 days	5.0175	89.0
		60 days	5.0096	34.9
		9 months	5.0842	< 20 (< LOQ)
	M8	7 days	5.0213	213.9
		15 days	4.9912	101.0
		30 days	5.0066	69.8
		45 days	5.0173	26.7
		60 days	5.0048	31.9
		9 months	5.0699	< 20 (< LOQ)

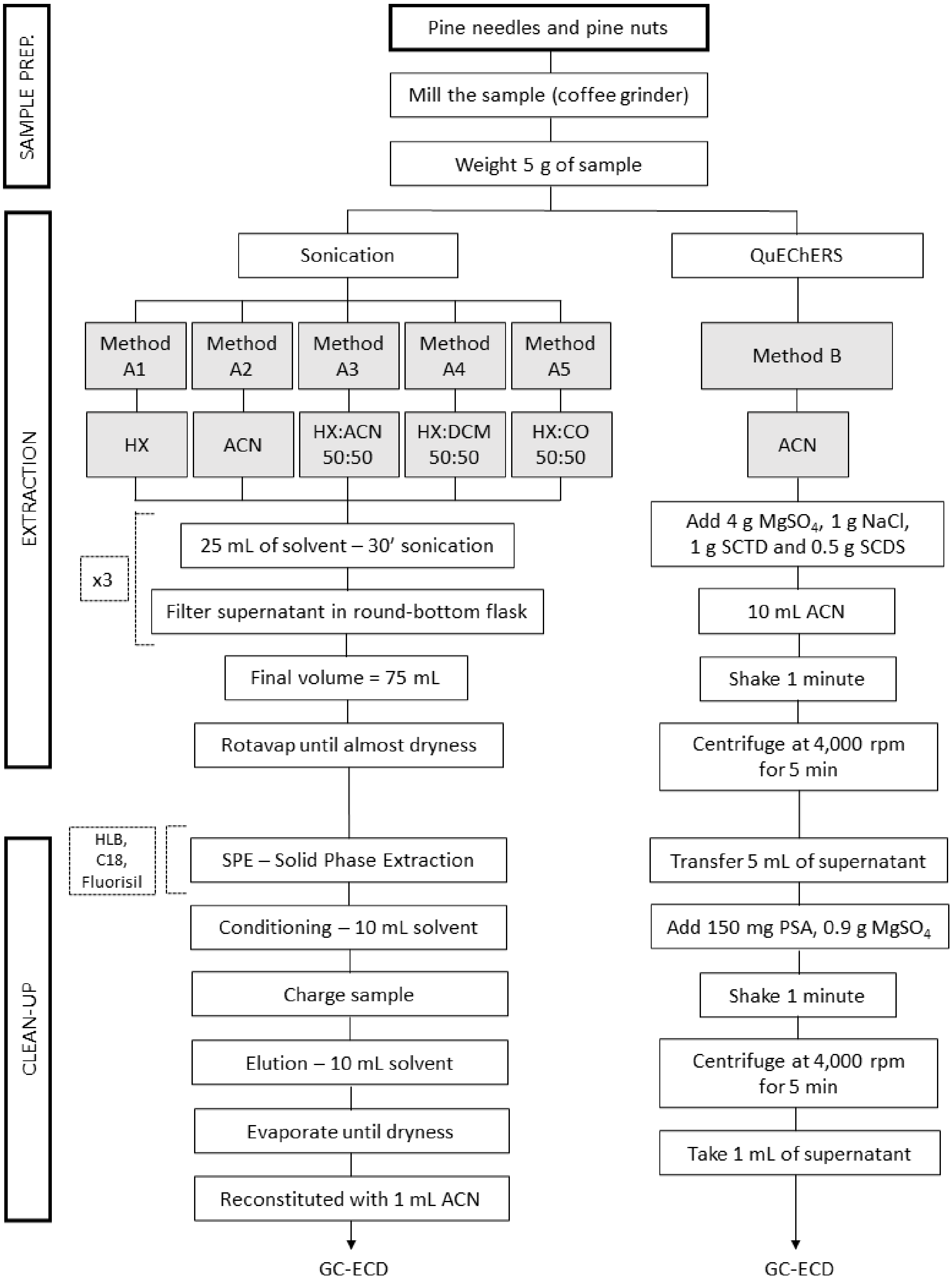

Figure S1. Detailed steps of the extraction and clean-up procedures under study.

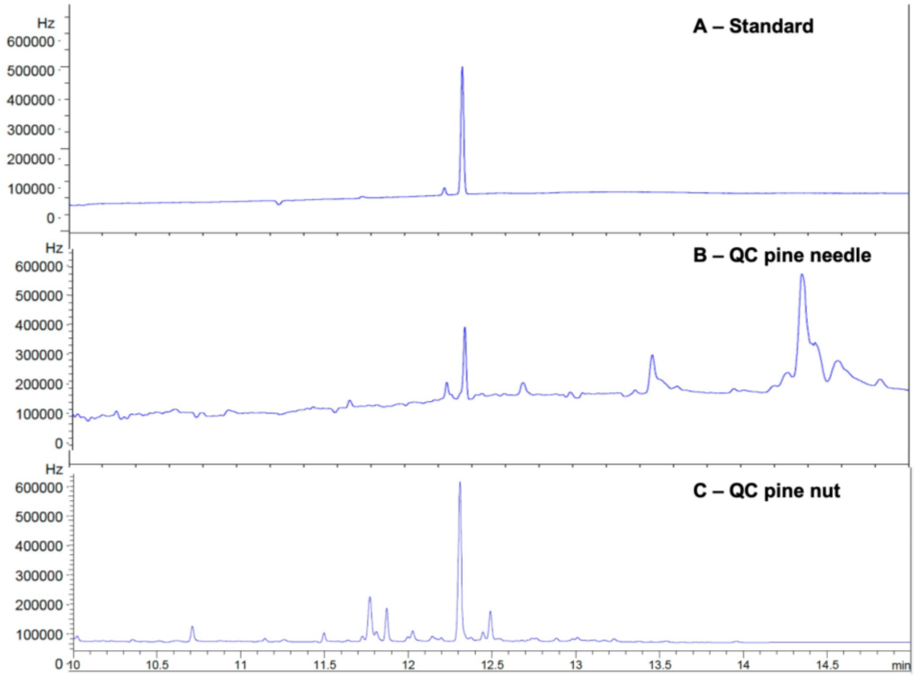

Figure S2. GC-ECD chromatogram of an standard at 20 µg kg^{-1} (A), an a QC of pine needle at 20 µg kg^{-1} (B) and pine nuts at 20 µg kg^{-1} (C).
